# Effects of ABCG2 dysfunction on hyperuricemia progression in premenopausal and postmenopausal women

**DOI:** 10.1007/s13577-026-01380-6

**Published:** 2026-06-18

**Authors:** Yuka Miyoshi, Akiyoshi Nakayama, Hiroshi Nakashima, Itsumi Hashimoto, Yusuke Kawamura, Seiko Shimizu, Yu Toyoda, Tomoko Mizuno, Risa Tanabe, Yoshinobu Hamada, Mako Nagayoshi, Yoko Kubo, Rieko Okada, Takashi Matsunaga, Noriyuki Yoshioka, Satoko Iwasawa, Miki Ueno, Kimiko Hayano, Hirofumi Nakaoka, Ken Yamamoto, Kenji Wakai, Nariyoshi Shinomiya, Hirotaka Matsuo

**Affiliations:** 1 Department of Preventive Medicine and Public Health, National Defense Medical College, 3-2 Namiki, Tokorozawa, Saitama 359-8513 Japan; 2 Research Division, Maritime Self-Defense Force Undersea Medical Center, Yokosuka, Japan; 3 Department of Integrative Physiology and Bio-nano Medicine, National Defense Medical College, 3-2 Namiki, Tokorozawa, Saitama 359-8513 Japan; 4 International Research Collaboration Officer, National Defense Medical College Research Institute, National Defense Medical College, 3-2 Namiki, Tokorozawa, Saitama 359-8513 Japan; 5 Division of Nursing, National Defense Medical College, 3-2 Namiki, Tokorozawa, Saitama 359-8513 Japan; 6 Department of Obstetrics and Gynecology, National Defense Medical College, 3-2 Namiki, Tokorozawa, Saitama 359-8513 Japan; 7 Department of Preventive Medicine, Nagoya University Graduate School of Medicine, Nagoya, Japan; 8 Department of Nursing, National Defense Medical College, 3-2 Namiki, Tokorozawa, Saitama 359-8513 Japan; 9 Department of Community Health Nursing, National Defense Medical College, 3-2 Namiki, Tokorozawa, Saitama 359-8513 Japan; 10 Department of Biomedical Data Science, Kagoshima University Graduate School of Medical and Dental Sciences, Kagoshima, Japan; 11 Department of Medical Biochemistry, Kurume University School of Medicine, Kurume, Fukuoka Japan; 12 Japan Institute for Health Security, National Institute of Infectious Diseases, Tokyo, Japan; 13 Department of Biomedical Information Management, National Defense Medical College Research Institute, National Defense Medical College, 3-2 Namiki, Tokorozawa, Saitama 359-8513 Japan

**Keywords:** *ABCG2* variant, Gout/hyperuricemia, Menopause, Genetic factors, Population attributable fractions (PAFs)

## Abstract

Serum uric acid (SUA) levels are influenced by environmental and genetic factors, such as sex and *ABCG2* variants. Their poorlyunderstood links to menstrual status prompted us to conduct this study to evaluate the effects of menstrual status-related ABCG2 function, detectable from the presence of its dysfunctional variants, on SUA levels. Recruited for the present study were 9244 Japanese individuals, comprising 4466 women and 4778 men. We evaluated the effect size on SUA and population attributable fractions (PAFs) for hyperuricemia progression based on factors, including ABCG2 function, BMI, alcohol consumption, age, and menstrual status (in women). The results, from the viewpoint of SUA increase, showed a 25% ABCG2 dysfunction to be equivalent to the effects of a 1.67 kg/m^2^ increase in BMI, alcohol consumption of 154.2 g/week, an 18.6-year increase in age, and, in women, 42.7% due to menopause. In the tested female sample, the PAF for hyperuricemia due to ABCG2 dysfunction (32.8%) exceeded those for overweight/obesity (19.5%) and aging (28.7%). Menopausal status exhibited the highest PAF at 63.0%. In premenopausal individuals, the PAF for ABCG2 dysfunction was 45.8%, much higher than that after menopause (29.6%). Although premenopausal women generally had a lower prevalence of hyperuricemia than postmenopausal individuals, those with dysfunctional variants of *ABCG2* tended to show a higher SUA increase and PAFs in premenopausal than in postmenopausal women. These results indicate that genetic testing might be helpful in preventing and treating hyperuricemia, particularly for those with higher SUA before menopause, in both individuals and in populations as a whole.

**Supplementary Information:**

The online version contains supplementary material available at 10.1007/s13577-026-01380-6.

## Introduction

Although hyperuricemia is a common disease in men, it is often neglected in women due to its low prevalence. However, high serum uric acid (SUA) levels in women are reported to raise the risk of total mortality [1] and negatively affect reproductive health: not only spontaneous pregnancy complications such as preeclampsia [2] but also assisted reproductive technology outcomes (pregnancy, live birth, and miscarriage) [3]. Moreover, SUA levels and the prevalence of gout increase after menopause [4, 5], making it imperative to conduct research on hyperuricemia in women both before and after menopause.

Environmental factors, such as obesity, alcohol consumption, and a purine-rich diet, are known to cause hyperuricemia, but recent studies also have revealed the significance of genetic factors [6]. Clinical genetic analysis, particularly genome-wide association studies (GWASs), have revealed numerous genes that are associated with gout and SUA [7–12]. These include membrane transporter genes [7, 9, 11], notably the ATP-binding cassette transporter G2 (*ABCG2*). *ABCG2* encodes a urate transporter located in the renal tubules and small intestinal epithelial cells [13]. Its common dysfunctional polymorphisms Q126X (rs72552713) and Q141K (rs2231142) are a strong risk factor for gout and hyperuricemia[14], as they reduce uric acid secretion from the kidney and small intestine[15]. The ABCG2 function can also be estimated by combining these two genetic variants [14, 16]. Furthermore, Asian populations, including the Japanese, are known to have dysfunctional ABCG2 more frequently than Caucasians [17].

The lack of studies reporting the effects of menopausal condition in comparison with those of ABCG2 dysfunction prompted us to embark on the present investigation, which was designed to elucidate the effects of ABCG2 dysfunction and other environmental factors on hyperuricemia progression as well as SUA levels before and after menopause in the Japanese population.

## Methods

### Study participants

Recruited for the present study were 9244 Japanese aged 35–69 years who had undergone health examinations. All the participants in this study were recruited from the Shizuoka and Daiko Areas of the Japan Multi-Institutional Collaborative Cohort Study (J-MICC Study) [18–20]. Written informed consent was obtained from all the participants. Data on their sex, age, alcohol consumption, and medications were acquired by self-administered questionnaire. Alcohol consumption was calculated based on the participants’ written questionnaires, as shown in Supplementary Table 1 [6].

Those who had used drugs that might affect female hormone levels or had undergone premenopausal oophorectomy or hysterectomy (excluding partial hysterectomy) were excluded from the study. Menopause was defined as the absence of regular menstruation for at least one year: information was obtained from responses to a self-administered questionnaire. Those who were under treatment for or who had past histories of gout/hyperuricemia were excluded from the analysis of SUA levels.

### Genetic analysis

Genomic DNA was extracted from the participants’ whole peripheral blood cells [21]. Genotyping of the two dysfunctional variants in the *ABCG2* gene, rs72552713 (c.376C>T, p.Q126X) and rs2231142(c.421C>A, p.Q141K), was performed using the TaqMan method (ThermoFisher Scientific, Waltham, MA, USA) with LightCycler 480 (Roche Diagnostics, Mannheim, Germany). Custom TaqMan assay probes were designed as follows. For p.Q126X, VIC-CCACTAATACTTACTTGTACCAC and FAM-CCACTAATACTTACTTATACCAC; for p.Q141K, VIC-CTGCTGAGAACTGTAAGTT and FAM-CTGCTGAGAACTTTAAGTT.

The participants were divided into four groups according to combination of common dysfunctional variants of *ABCG2*, non-functional Q126X, and half functional Q141K, as follows [6]: full function (normal function), 3/4 function (mild dysfunction), 1/2 function (moderate dysfunction), and ≤ 1/4 function (severe dysfunction).

### Statistical analysis

All the statistical analyses were performed using R (v.4.1.1) and SAS (v9.4; Cary, NC, USA) software. Those under treatment for or who had past histories of gout/hyperuricemia were excluded; and mean SUA levels, body mass index (BMI), alcohol consumption, and age were calculated. Comparisons of SUA levels between premenopausal and postmenopausal women were made using Student’s *t* test. Single regression analyses were performed with SUA levels as the objective variable and ABCG2 function as the explanatory variable. Multiple regression analyses were conducted using the method of decreasing variables, with SUA levels as the objective variable and ABCG2 function, age, BMI, and alcohol consumption as explanatory variables. In women, menopause was added to the model as an explanatory variable. The effect sizes on SUA levels for each factor were compared using regression coefficients (β). The regression coefficients of ABCG2 function in premenopausal and postmenopausal women were also assessed by tests of interaction. The ratio of regression coefficients (β_ABCG2_/β: effect size on SUA levels by a 25% decrease in ABCG2 dysfunction vs. by each risk factor) was calculated to compare the effect of ABCG2 dysfunction on increased SUA levels in individuals subject to other environmental factors.

To evaluate the percentage of hyperuricemic patients originated from ABCG2 dysfunction, we calculated the population attributable fraction (PAF) of ABCG2 dysfunction for hyperuricemia progression. PAF refers to the proportion of cases or deaths attributable to a specific risk factor. For example, “the PAF of ABCG2 dysfunction for hyperuricemia is 30%” means that “30% of patients developed hyperuricemia due to ABCG2 dysfunction.” Hyperuricemia in both men and women has been defined as having an SUA level of > 7.0 mg/dl (420 µmol/l). For women, the threshold for SUA level was also set at 6.0 mg/dl (360 µmol/l) if indicated, as a previous study [1] has reported an increased risk of total mortality at 6.0 mg/dl and above. Those under treatment for or with past histories of gout or hyperuricemia were also included as being hyperuricemic. The criteria for being at risk for each of the known factors affecting SUA levels were as follows [6]: ABCG2 dysfunction was defined as ABCG2 function being less than full. Overweight/obesity was defined as a BMI of 25 kg/m^2^ or more. Heavy drinking was defined as alcohol consumption that exceeded 196 g/week of pure ethanol for men and 98 g/week of pure ethanol for women. Aging was defined as being 60 years and over.

The PAF of ABCG2 function and other typical risk factors for hyperuricemia was calculated using the following equation [6]:

$${\mathrm{PAF}}\, = \,\left[ {\{ \left( {N_{{{\mathrm{HUA}},{\mathrm{Risk}}}} /N_{{{\mathrm{Risk}}}} {-}N_{{{\mathrm{HUA}},{\mathrm{NonRisk}}}} /N_{{{\mathrm{NonRisk}}}} } \right)\, \times \,\left( {N_{{{\mathrm{Risk}}}} /N_{{{\mathrm{All}}}} } \right)\} /\left( {N_{{{\mathrm{HUA}}}} /N_{{{\mathrm{All}}}} } \right)} \right]\, \times \,{1}00.$$

(“*N*_HUA,Risk_” and “*N*_HUA,NonRisk_”, respectively, indicate the numbers of hyperuricemia(HUA) patients in the risk group and the non-risk group. “*N*_Risk_” and “*N*_NonRisk_”, respectively, represent the numbers of individuals in the risk group and non-risk group. “*N*_HUA_” and “*N*_All_”, respectively, mean the number of all hyperuricemia patients and all participants.)

To improve statistical robustness, random resampling methods using computer simulations are often applied. In this study, to evaluate the 95% confidence interval (95% CI) of PAF, the bootstrap method [22] was used for random resampling of all participants’ data sets, with 10,000 replacements.

### Ethical considerations

This study was approved by the Institutional Ethical Committee (National Defense Medical College and Nagoya University), and was performed in accordance with the Declaration of Helsinki.

## Results

**Fig. 1:**
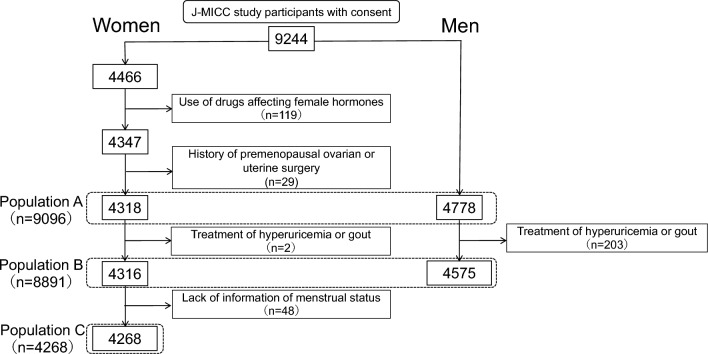
Subjects of this study. 9096 participants (Population A) were selected for the present study. For the analyses of serum uric acid levels of women and men, population B was used. For those of pre- and postmenopausal women, population C was used

Figure 1 shows the selection of subjects constituting the 9244 participants (4466 women and 4778 men). From 4,466 women, we excluded 119 women who were using drugs that affect female hormones and 29 women with a history of premenopausal oophorectomy/hysterectomy. A total of 9096 participants (4318 women and 4778 men: Population A) were included in the risk ratio (RR) and PAF evaluations for progression of hyperuricemia. After excluding two women and 203 men who were undergoing treatment for gout or hyperuricemia, 8891 subjects (4316 women and 4575 men: Population B) were then evaluated for SUA levels. Regression analysis was performed on Population B separately for men and for women and on 4268 women (Population C), and then separately for pre- and postmenopause individuals, having excluded 48 whose menstrual status could not be ascertained from the 4316 women in Population B.

ABCG2 dysfunction (≤ 1/4 function) was observed in 53.5% of the women and 53.1% of the men (Population A). The MAFs of Q126X and Q141K were 0.0217 and 0.298, respectively, which were similar to previously reported values [14, 17, 23]. Both variants were in Hardy–Weinberg equilibrium (*p* > 0.05).

### Effects of menstrual status and sex on elevated SUA levels

**Fig. 2:**
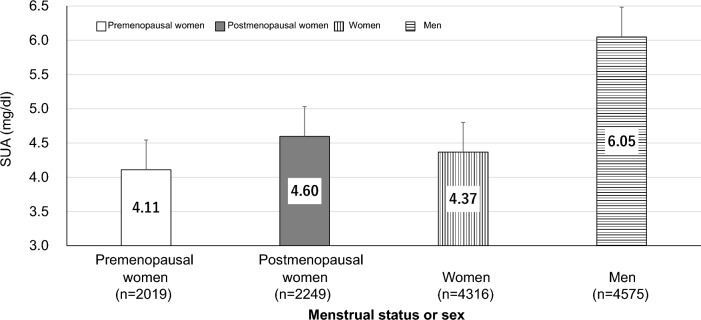
Serum uric acid (SUA) levels of pre- and postmenopausal women. SUA levels of pre- and postmenopausal women were obtained from population C (*n* = 4268). Those of women and men were also from population B (*n* = 8891). SUA is expressed as mean + standard error

The characteristics of the participants are shown in Supplementary Table 2. The mean SUA level was significantly higher in postmenopausal than in premenopausal women (*p* < 0.0001), with an SUA level of 4.11 mg/dl in premenopausal women compared with 4.60 mg/dl in postmenopausal women. The mean SUA level was 4.37 mg/dl in women and 6.05 mg/dl in men (Fig. 2).

**Fig. 3:**
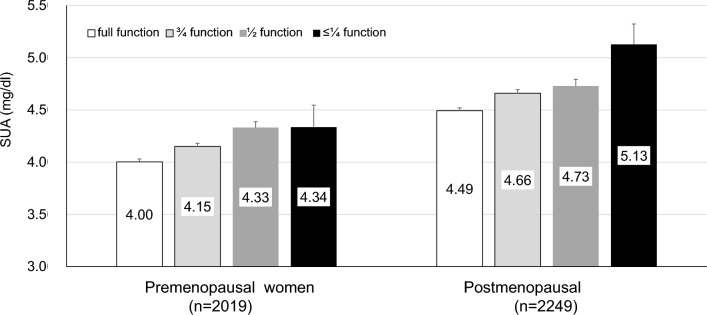
Effects of ABCG2 function on serum uric acid (SUA) levels of pre- and postmenopausal women. SUA levels according to each ABCG2 function were shown for pre- and postmenopausal women (population C). SUA is expressed as mean + standard error. Referred from [25] in the main text

The mean SUA levels for premenopausal and postmenopausal women and men, grouped according to ABCG2 function, are shown in Fig. 3 and Supplementary Table 3. SUA levels increased significantly with ABCG2 dysfunction in both premenopausal and postmenopausal women by single regression analysis (*p* < 0.0001). SUA levels for each ABCG2 function were higher in postmenopausal women than in premenopausal women (Supplementary Fig 1).

### Effect of each risk factor on elevated SUA levels

To evaluate the effect size on SUA for each factor, 8891 individuals (4316 women and 4575 men: Population B), who had received no treatment for gout and/or hyperuricemia, were selected from 9096 participants (Population A), and further regression analysis was performed. The results of multiple regression analysis with SUA levels and ABCG2 dysfunction and known environmental factors are shown in Table 1. ABCG2 function, BMI, alcohol consumption, and age were all shown to be significant positive explanatory factors in both premenopausal and postmenopausal women. Menopause was also a significant positive explanatory factor. In men, all factors were significant explanatory factors, but only age was a negative factor.

**Table 1:** Effects of ABCG2 function and other risk factors on serum uric acid levels

Population	Risk factor^a^	*β*^b^ (regression coefficient)	95% CI	*P* value	*β*_ABCG2_/*β* (ratio of regression coefficient)
Women	ABCG2 function	0.148	0.111–0.184	< 0.0001	1
(*n* = 4316)	BMI (kg/m^2^)	0.0886	0.0796–0.0976	< 0.0001	1.67
	Alcohol consumption (g/week of pure alcohol)	9.57 × 10^–4^	7.17 × 10^–4^–1.20 × 10^–3^	< 0.0001	154.2
	Age (years)	7.93 × 10^–3^	3.13 × 10^–3^–0.0127	0.0012	18.6
	Menopausal status	0.346	0.253–0.440	< 0.0001	0.427
Premenopausal	ABCG2 function	0.163	0.114–0.213	< 0.0001	1
(*n* = 2019)	BMI (kg/m^2^)	0.0804	0.0688–0.0919	< 0.0001	2.03
	Alcohol consumption (g/week of pure alcohol)	7.21 × 10^–4^	4.40 × 10^–4^–1.00 × 10^–3^	< 0.0001	226.1
	Age (years)	7.10 × 10^–3^	2.95 × 10^–4^–0.0139	0.0411	23.0
Postmenopausal	ABCG2 function	0.132	0.0785–0.186	< 0.0001	1
(*n* = 2249)	BMI (kg/m^2^)	0.0977	0.0839–0.111	< 0.0001	1.35
	Alcohol consumption (g/week of pure alcohol)	1.35 × 10^–3^	9.34 × 10^–4^–1.77 × 10^–3^	< 0.0001	97.8
	Age (years)	9.29 × 10^–3^	2.52 × 10^–3^–0.0161	0.0071	14.2
Men	ABCG2 function	0.212	0.164–0.259	< 0.0001	1
(*n* = 4575)	BMI (kg/m^2^)	0.0914	0.0793–0.103	< 0.0001	2.32
	Alcohol consumption (g/week of pure alcohol)	4.85 × 10^–4^	3.37 × 10^–4^–6.33 × 10^–4^	< 0.0001	436.4
	Age (years)	–5.08 × 10^–3^	–8.84 × 10^–3^– –1.32 × 10^–3^	0.0083	–41.7

For women and men, multiple regression analyses were performed on population B. For pre- and postmenopausal women, those were performed on population C

*BMI* body mass index

^a^Calculation for ABCG2 function was conducted for full function as 1, 3/4 function (mild dysfunction) as 2, 1/2 function (moderate dysfunction) as 3, and 1/4 function (severe dysfunction) as 4. Calculation for menopausal status was conducted for premenopausal women as 1 and postmenopausal women as 2

^b^“*β*” indicates the increase of serum uric acid (mg/dl) per unit of each risk factor. The ratio of regression coefficients (*β*_ABCG2_/*β*) was calculated from the *β* of ABCG2 function divided by that of each risk factor, showing an effect equivalent to a 1/4 decrease in ABCG2 function in terms of ability to increase serum uric acid levels

The effect size on SUA, i.e., the regression coefficient (β) for a 25% decrease in ABCG2 dysfunction was a gain of 0.148 mg/dl, whereas the effects of other environmental factors were as follows: 0.0886 mg/dl per BMI unit, 9.57 × 10^–4^ mg/dl per gram of pure alcohol consumed per week, 7.93 × 10^–3^ mg/dl per year in age, and 0.346 mg/dl between menopausal and non-menopausal status in women.

Further calculations by menstrual status showed that all factors significantly increased SUA levels in both premenopausal and postmenopausal women. The β for a 25% decrease in ABCG2 dysfunction was a gain of 0.163 mg/dl in premenopausal and 0.132 mg/dl in postmenopausal women.

### Effect of each risk factor corresponding to ABCG2 dysfunction

**Fig. 4:**
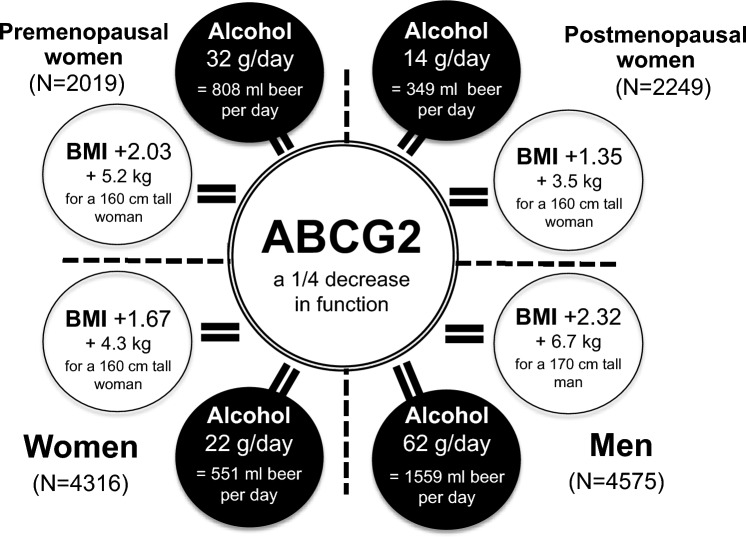
Comparison of factors equivalent to a 1/4 decrease in ABCG2 function in terms of ability to increase serum uric acid levels. *BMI* body mass index

From the results of multiple regression analysis, the degree of increase of each factor causing an increase in SUA levels equivalent to a 25% decrease in ABCG2 dysfunction in an individual, i.e., the ratio of the regression coefficients (*β*_ABCG2_/*β*), was calculated (Table 1, Fig. 4). ABCG2 dysfunction had a stronger effect than other environmental factors: a 25% decrease in ABCG2 dysfunction showed an effect equivalent to an increase of BMI by 1.67 points, 154.2 g/week alcohol intake as pure alcohol, aging by 18.6 years, or 0.427 times menstrual status in terms of the tendency to increase SUA levels in women. In premenopausal women, a 25% decrease in ABCG2 dysfunction showed an effect equivalent to a 2.03-point increase in BMI, 226.1 g/week alcohol intake, or an age increase of 14.2 years; and in postmenopausal women, a 25% decrease in ABCG2 dysfunction showed an effect equivalent to a 1.35-point increase in BMI, 97.8 g/week alcohol intake, or aging by 23.0 years in terms of tendency to increase SUA levels. The ratio of regression coefficients was higher in premenopausal women for all factors.

### Genetic risk factor for hyperuricemia in the general population

To compare the influence of each factor on hyperuricemia progression, PAFs were calculated for the general population in addition to RR for individuals (Table 2, Supplementary Fig 2). Note that the RR and PAF for aging were not calculated for premenopausal women, as there were no subjects aged 60 years or older.

**Table 2:** PAF and risk ratio of each risk factor for hyperuricemia progression

Population^a^	Risk factor^b^	PAF	95% CI	Risk ratio	95% CI	*P* value
Women	**ABCG2 dysfunction**	**32.8**	**19.5–46.2**	**1.91**	**1.44–2.55**	**6.16 × 10**^**–6**^
(*n* = 4318)	**Overweight/obesity**	**19.5**	**12.8–26.4**	**3.08**	**2.32–4.10**	**2.02 × 10**^**–15**^
	Heavy drinking	–1.03	–7.52–5.83	0.95	0.67–1.34	0.758
	**Aging**	**28.7**	**19.7–37.7**	**2.52**	**1.94–3.28**	**1.56 × 10**^**–12**^
	**Menopausal status**	**63.0**	**52.4–73.8**	**4.24**	**2.97–6.04**	**1.58 × 10**^**–18**^
Premenopausal	**ABCG2 dysfunction**	**45.8**	**14.3–75.3**	**2.57**	**1.22–5.44**	**0.0102**
(*n* = 2019)	**Overweight/obesity**	**25.1**	**8.24–43.0**	**4.07**	**2.06–8.02**	**1.41 × 10**^**–5**^
	Heavy drinking	–2.09	–20.4–17.4	0.92	0.42–2.00	0.826
	Aging	–	–	–	–	–
Postmenopausal	**ABCG2 dysfunction**	**29.6**	**15.0–44.0**	**1.79**	**1.31–2.44**	**1.69 × 10**^**–4**^
(*n* = 2251)	**Overweight/obesity**	**17.8**	**10.5–25.1**	**2.80**	**2.05–3.82**	**6.60 × 10**^**–11**^
	Heavy drinking	3.50	–3.00–10.7	1.25	0.85–1.83	0.260
	**Aging**	**15.4**	**0.62–30.2**	**1.36**	**1.01–1.82**	**0.0395**
Men	**ABCG2 dysfunction**	**30.2**	**25.0–35.5**	**1.81**	**1.62–2.03**	**9.72 × 10**^**–27**^
(*n* = 4778)	**Overweight/obesity**	**14.9**	**11.7–18.2**	**1.69**	**1.52–1.87**	**6.92 × 10**^**–22**^
	**Heavy drinking**	**10.4**	**6.39–14.2**	**1.34**	**1.20–1.49**	**1.22 × 10**^**–7**^
	Aging	1.89	–1.24–5.16	1.07	0.96–1.20	0.241

For women and men, PAF and risk ratio were calculated in population A

For pre- and postmenopausal women, those were calculated in 4270 women whose menstrual status was known among population A

Significant factors are shown in bold

*PAF* population attributable fraction, *CI* confidence interval

^a^For women, hyperuricemia was defined when their serum uric acid levels were over 6 mg/dl; for men, hyperuricemia was defined when their serum uric acid levels were over 7 mg/dl

^b^Each risk factor was defined as follows: ABCG2 dysfunction, having dysfunctional variants of *ABCG2* (Q126X and/or Q141K); aging, ≥ 60 years; heavy drinking, alcohol consumption > 98 g/week (women) and 196 g/week (men) of pure alcohol; overweight/obesity, body mass index (BMI) ≥ 25.0 kg/m^2^; sex, men. PAF and risk ratio for aging were not calculated for premenopausal women, because there were no subjects older than 60 years old

First, regarding RR for hyperuricemia, three factors were significant in women, the four factors ABCG2 dysfunction, overweight/obesity, aging, and menopause were significant, with values of 1.91, 3.08, 2.52, and 4.24, respectively. In postmenopausal women, ABCG2 dysfunction, overweight/obesity, and aging were significant, with values of 1.79, 2.80, and 1.36. In premenopausal women, ABCG2 dysfunction and obesity were significant, with values of 2.57 and 4.07, which were higher than those of postmenopausal women. In men, the three factors ABCG2 dysfunction, overweight/obesity, and heavy drinking were significant, with values of 1.81, 1.69, and 1.34, respectively. No significant values were obtained for the RR of heavy drinking in premenopausal and postmenopausal women, or for the RR of aging in men.

The PAF for ABCG2 dysfunction was 32.8% in 4,318 women, higher than the 19.5% for obesity and 28.7% for aging, followed by the 63.0% for menopause. Three environmental factors were significant in postmenopausal women: ABCG2 dysfunction, overweight/obesity, and aging, with ABCG2 dysfunction having a value of 29.6%, significantly higher than the 17.8% for obesity and 15.4% for aging. In contrast, among premenopausal women, the two factors of ABCG2 dysfunction and overweight/obesity predominated, with the PAF for ABCG2 dysfunction (45.8%) almost double that of 25.1% for obesity. Among the 4778 men, the PAF for ABCG2 dysfunction was the highest at 30.2%, followed by the factors of overweight/obesity (14.9%) and heavy drinking (10.4%). As in the RR and PAF, no significant values were obtained for heavy drinking in either premenopausal or postmenopausal women or for aging in men.

## Discussion

The present study revealed that ABCG2 dysfunction, estimated based on two common dysfunctional *ABCG2* polymorphisms (Q126X and Q141K), affects SUA levels independently of typical environmental factors in both premenopausal and postmenopausal women in the Japanese population. It was also found that the effect of ABCG2 dysfunction on progression of hyperuricemia exceeded that of all environmental factors.

In women, mean SUA levels were significantly higher in postmenopausal than in premenopausal women. The increased prevalence of hyperuricemia in women after menopause indicated a decreased effect of female hormones on SUA levels, likely because female hormones are reported to promote uric acid excretion [24]. Nevertheless, whereas it was expected that the SUA of postmenopausal women would be close that of men, the present study revealed that the SUA of postmenopausal women was between that of premenopausal women and men and relatively close to that of premenopausal women. This result could be explained not only by decreased female hormone levels in postmenopausal women, but also to male hormones in the male population.

The results of stratified analyses of men and women in our previous studies indicate that the influence of each factor on SUA levels in the population differs between men and women [25]. The present study showed that individual factors, including *ABCG2* variants, have different effects on SUA levels, even between premenopausal and postmenopausal women. For example, women with ABCG2 dysfunction had higher SUA than those with normal ABCG2 function, both before and after menopause. Although high SUA in premenopausal women is relatively rare, ABCG2 dysfunction has stronger effects on premenopausal women (*β* = 0.163 mg/dl for SUA) than on postmenopausal women (*β* = 0.132 mg/dl). The difference of 0.03 mg/dl per each quarter of ABCG2 dysfunction is significant, considering that it is 0.06 between the sexes. Since hyperuricemic females are reported to be at risk of eclampsia [2], preventing progression of hyperuricemia is important in young women, who tend not to be tested for this condition. Moreover, SUA levels were higher in women with ABCG2 dysfunction than those without it. SUA levels have a relationship with total mortality [1], making it worthwhile to focus on ABCG2 dysfunction to ensure lifelong risk management.

The ratio of the regression coefficients (*β*_ABCG2_/*β*) represents how much of an impact a 25% decrease in ABCG2 dysfunction is likely to have compared to other risk factors. The ratio of regression coefficients was also higher in premenopausal than in postmenopausal women for all factors, indicating the impact of ABCG2 dysfunction compared to other factors to be greater in premenopausal than in postmenopausal women. Furthermore, the comparable impact among genetic and typical environmental effects indicates that the innate burden exerted by genetic effects could be convertible to environmental effects by reducing their weight and/or alcohol intake: for example, making it a trigger for behavioral change on the part of patients [25]. In premenopausal and postmenopausal women, the quantity of alcohol consumed corresponding to a quarter reduction in ABCG2 function is 226.1 g/week (e.g., 2.3 × 350-ml cans of beer per day) and 97.8 g/week (e.g., one 350-ml can of beer per day), respectively. These amounts could be feasible management targets as lifestyle modifications. BMI also corresponded with an increase of 2.03 kg/m^2^ (5.2 kg for 160 cm-tall premenopausal women) and 1.35 kg/m^2^ (3.5 kg for 160 cm-tall postmenopausal women), both of which are feasible management goals. The results of this study suggest that even people with impaired ABCG2 function can prevent hyperuricemia through individual efforts. We have previously reported that the effect of each factor on SUA levels varies according to sex and ABCG2 function, and recommended setting more individualized guidance goals [25]. The present study is significant in the field of preventive medicine in that it makes it possible to suggest specific targets to attain. It would be clinically significant if a longitudinal study could be conducted to verify whether patient guidance that actually presents this goal of effort can promote behavior changes in patients and prevent the onset of hyperuricemia and gout.

A PAF represents the effects of risk factors in the population, and indicates the populational burden exerted by each factor. The PAF results for overweight/obesity do not differ significantly among men, premenopausal women, and postmenopausal women. This indicates that prevention by reducing body weight has similar effects in both men and women, irrespective of menstrual state. The PAFs for ABCG2 dysfunction were similar in men and women. However, there was a significant difference between premenopausal (45.8%) and postmenopausal (29.6%) women, indicating the importance of genotyping for *ABCG2* in premenopausal women to prevent progression of hyperuricemia. The PAFs of 45.8% and 29.6% for *ABCG2* reveal that the 45.8% and 29.6% prevalence of hyperuricemia patients in premenopausal and postmenopausal women, respectively, were caused by *ABCG2* dysfunction. These figures display the major impact of *ABCG2* dysfunction on hyperuricemia, especially in the context of the PAFs of smoking on cancer mortality (26.8%) and incidence (25.3%) in Japanese women [26]. Prevention of gout in the younger generation may also lead to the prevention of future cardiovascular disorders, making the results of this study useful as a basis for effective interventions in the general population.

In a previous study by Zhang et al. using four populations in the US [27], in which only Q141K was analyzed as an *ABCG2* variant, it was found that an increase of one minor allele A in Q141K increased SUA levels to a greater extent in postmenopausal than in premenopausal women. The discrepancy with our study might be explained by the following three possible mechanisms. First, there are differences in the prevalence of hyperuricemia [28] and the influence of female hormones. The present study had excluded the effects of exogenous female hormones by applying two exclusion criteria: drug use and history of surgery in both pre- and postmenopausal women, whereas the previous study did not. Second, environmental factors, such as alcohol consumption, purine intake from food, and the prevalence of obesity, differ considerably between Japan and USA [29]. Third, there are ethnic differences in the prevalence of *ABCG2* gene polymorphism [17].

There are two limitations to the present study. First, only a few of the female participants were heavy drinkers. Only one premenopausal woman and 10 postmenopausal women engaged in hazardous drinking and had hyperuricemia as hyperuricemia was defined as having an SUA level of > 7.0 mg/dl. Even if an SUA level was set at 6.0 mg/dl, eight premenopausal women and 29 postmenopausal women were in hazardous drinking and hyperuricemia. The results of multiple regression analysis showed women’s alcohol consumption to be a significant explanatory factor for SUA levels, but the calculations of RR and PAF showed no significant values for heavy drinking in women. This may be due to the few heavy drinkers and low alcohol consumption by women (Supplementary Table 2), whereas heavy drinking was analyzed as a risk factor for the development of hyperuricemia in the calculations of RR and PAF.

Second, this was a cross-sectional study, which might explain why a negative regression coefficient for age on SUA levels in men and no significant values for aging were obtained in men in the RR and PAF calculations. SUA levels at the same age were reportedly higher in the Japanese than in the US working population, especially in the younger generations [30]. To prevent hyperuricemia and gout, longitudinal studies are necessary for accurate evaluation of each factor before and after menopause.

In summary, the effect of ABCG2 dysfunction on SUA levels was revealed to be greater than that of environmental factors, and to be unrelated to typical environmental factors. The influence of ABCG2 dysfunction on SUA levels was significant in both premenopausal and postmenopausal women, particularly in premenopausal individuals. This means that premenopausal women with hyperuricemia are more likely to have ABCG2 dysfunction. The results of genetic testing should not only provide useful information on the prognosis of hyperuricemia that will help to proactively improve lifestyles, especially those of premenopausal women, but also might be used to motivate people to improve their lifestyles even before the onset of hyperuricemia. We believe that this study will assist with the creation of genome-personalized goals that both promote behavior change in patients and prevent the progression of hyperuricemia and gout in both pre- and postmenopausal women.

## Supplementary Information

Below is the link to the electronic supplementary material.

Supplementary file1 (PPTX 1513 KB)

## Data Availability

Data are available upon reasonable request to the corresponding author.
