# Supplementary material for: Effects of ABCG2 dysfunction on hyperuricemia progression in premenopausal and postmenopausal women
Source: Hum Cell. 2026 Jun 18;39(7):88. doi: 10.1007/s13577-026-01380-6 (PMC13275769; doi:10.1007/s13577-026-01380-6)
Supplement: Supplementary file 1 — Supplementary file1 (PPTX 1513 KB) [file 13577_2026_1380_MOESM1_ESM.pptx]

## Slide 1
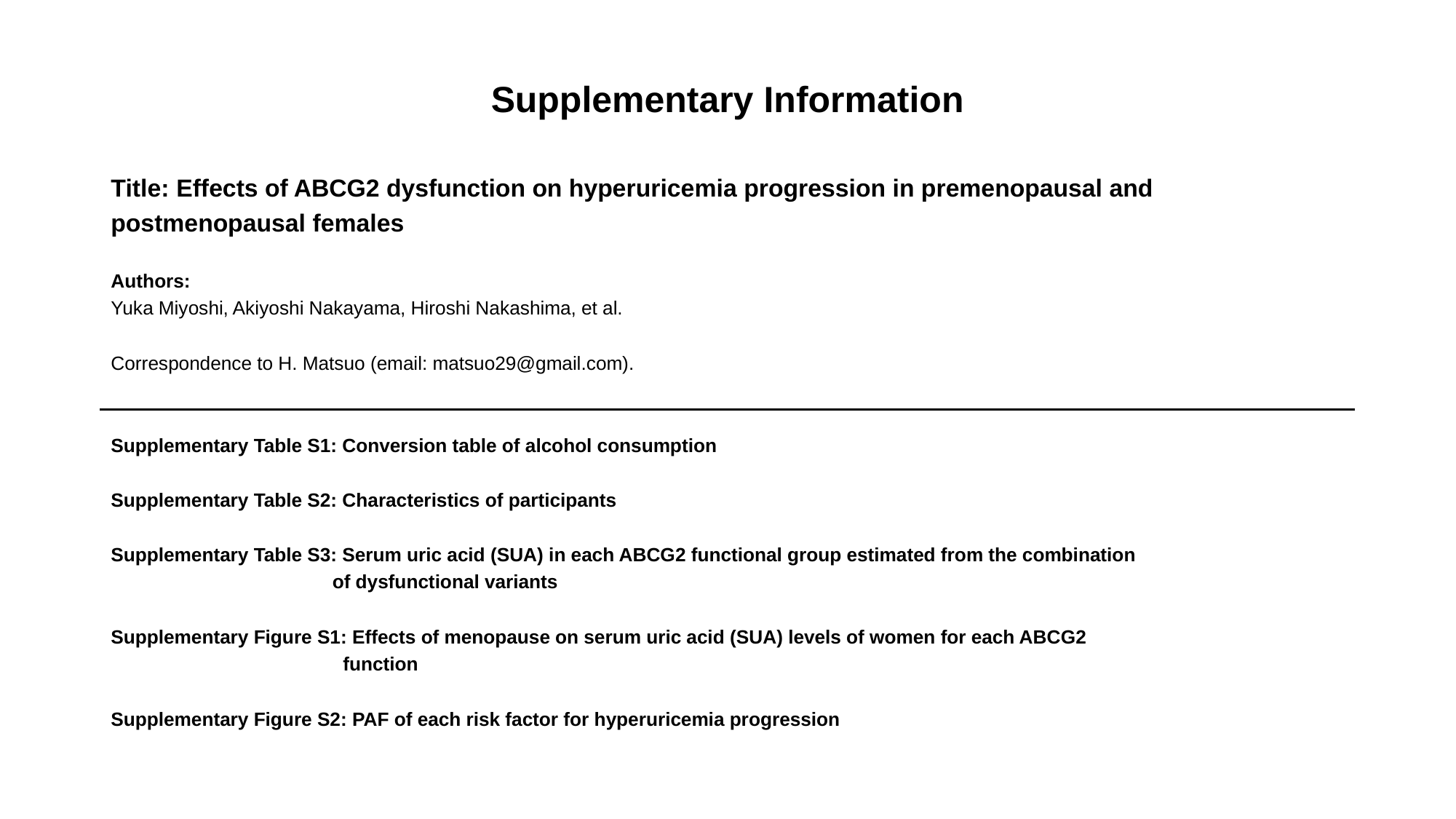

# Supplementary Information
Title: Effects of ABCG2 dysfunction on hyperuricemia progression in premenopausal and postmenopausal females
Authors:
Yuka Miyoshi, Akiyoshi Nakayama, Hiroshi Nakashima, et al.
Correspondence to H. Matsuo (email: matsuo29@gmail.com).
Supplementary Table S1: Conversion table of alcohol consumption
Supplementary Table S2: Characteristics of participants
Supplementary Table S3: Serum uric acid (SUA) in each ABCG2 functional group estimated from the combination
 of dysfunctional variants
Supplementary Figure S1: Effects of menopause on serum uric acid (SUA) levels of women for each ABCG2
 function
Supplementary Figure S2: PAF of each risk factor for hyperuricemia progression

## Slide 2
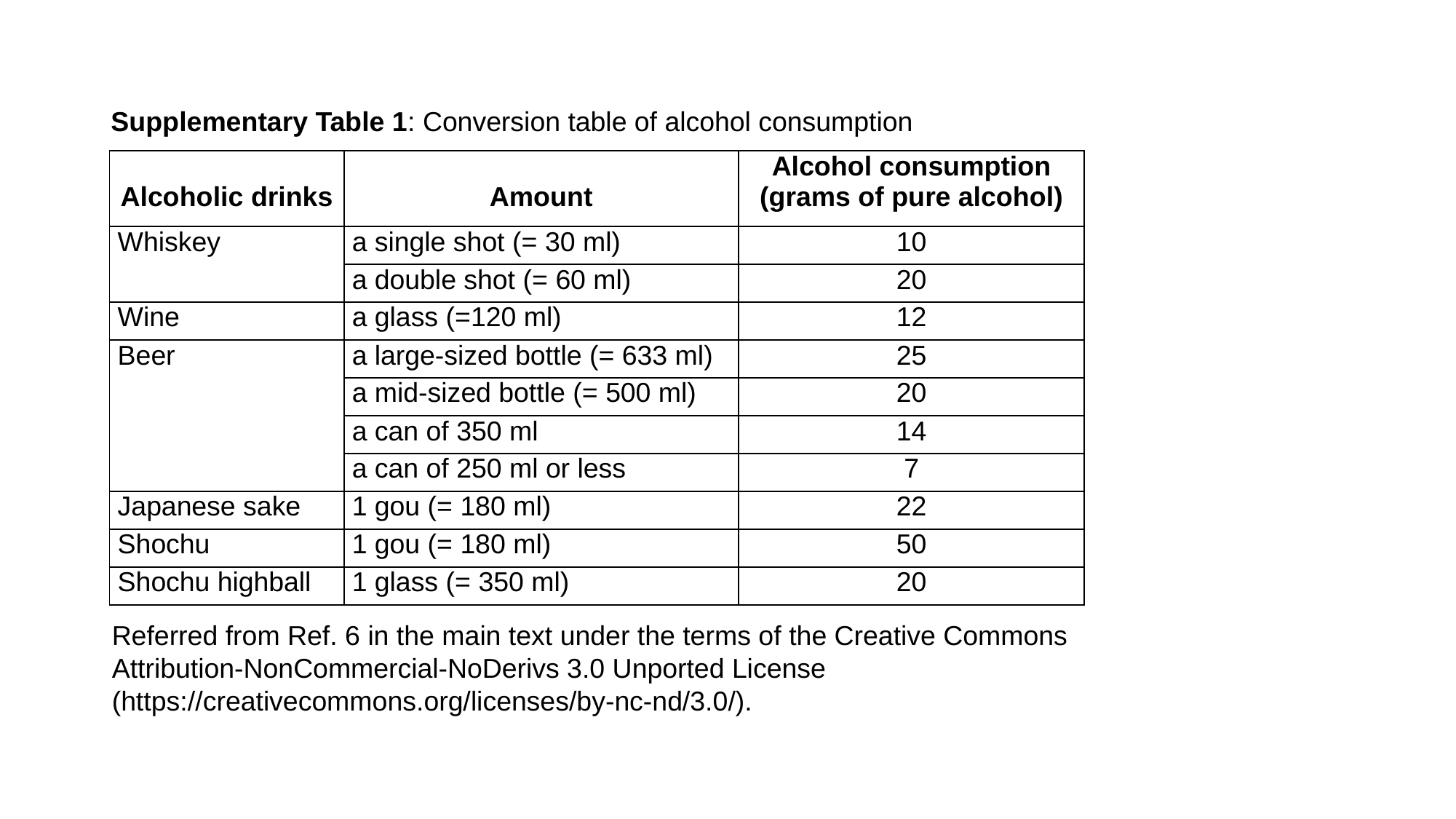

# Supplementary Table 1: Conversion table of alcohol consumption
| Alcoholic drinks | Amount | Alcohol consumption (grams of pure alcohol) |
| --- | --- | --- |
| Whiskey | a single shot (= 30 ml) | 10 |
| | a double shot (= 60 ml) | 20 |
| Wine | a glass (=120 ml) | 12 |
| Beer | a large-sized bottle (= 633 ml) | 25 |
| | a mid-sized bottle (= 500 ml) | 20 |
| | a can of 350 ml | 14 |
| | a can of 250 ml or less | 7 |
| Japanese sake | 1 gou (= 180 ml) | 22 |
| Shochu | 1 gou (= 180 ml) | 50 |
| Shochu highball | 1 glass (= 350 ml) | 20 |
Referred from Ref. 6 in the main text under the terms of the Creative Commons Attribution-NonCommercial-NoDerivs 3.0 Unported License (https://creativecommons.org/licenses/by-nc-nd/3.0/).

## Slide 3
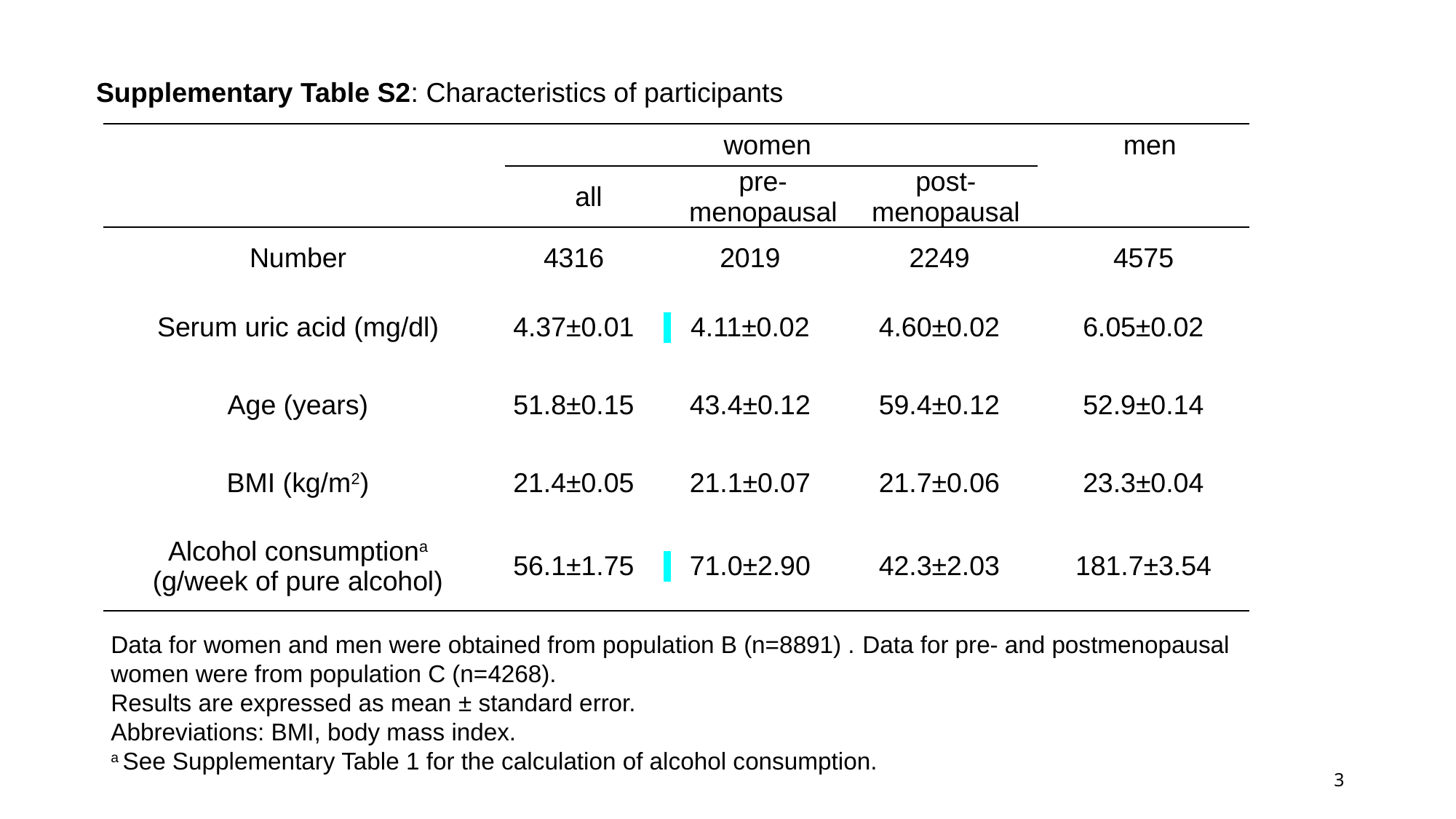

# Supplementary Table S2: Characteristics of participants
| | | women | | | | 閉経後女性 | | men |
| --- | --- | --- | --- | --- | --- | --- | --- | --- |
| | | all | | pre-menopausal | | post-menopausal | | |
| Number | | 4316 | | 2019 | | 2249 | | 4575 |
| Serum uric acid (mg/dl) | | 4.37±0.01 | | 4.11±0.02 | | 4.60±0.02 | | 6.05±0.02 |
| Age (years) | | 51.8±0.15 | | 43.4±0.12 | | 59.4±0.12 | | 52.9±0.14 |
| BMI (kg/m2) | | 21.4±0.05 | | 21.1±0.07 | | 21.7±0.06 | | 23.3±0.04 |
| Alcohol consumptiona (g/week of pure alcohol) | | 56.1±1.75 | | 71.0±2.90 | | 42.3±2.03 | | 181.7±3.54 |
Data for women and men were obtained from population B (n=8891) . Data for pre- and postmenopausal women were from population C (n=4268).
Results are expressed as mean ± standard error.
Abbreviations: BMI, body mass index.
a See Supplementary Table 1 for the calculation of alcohol consumption.
3

## Slide 4
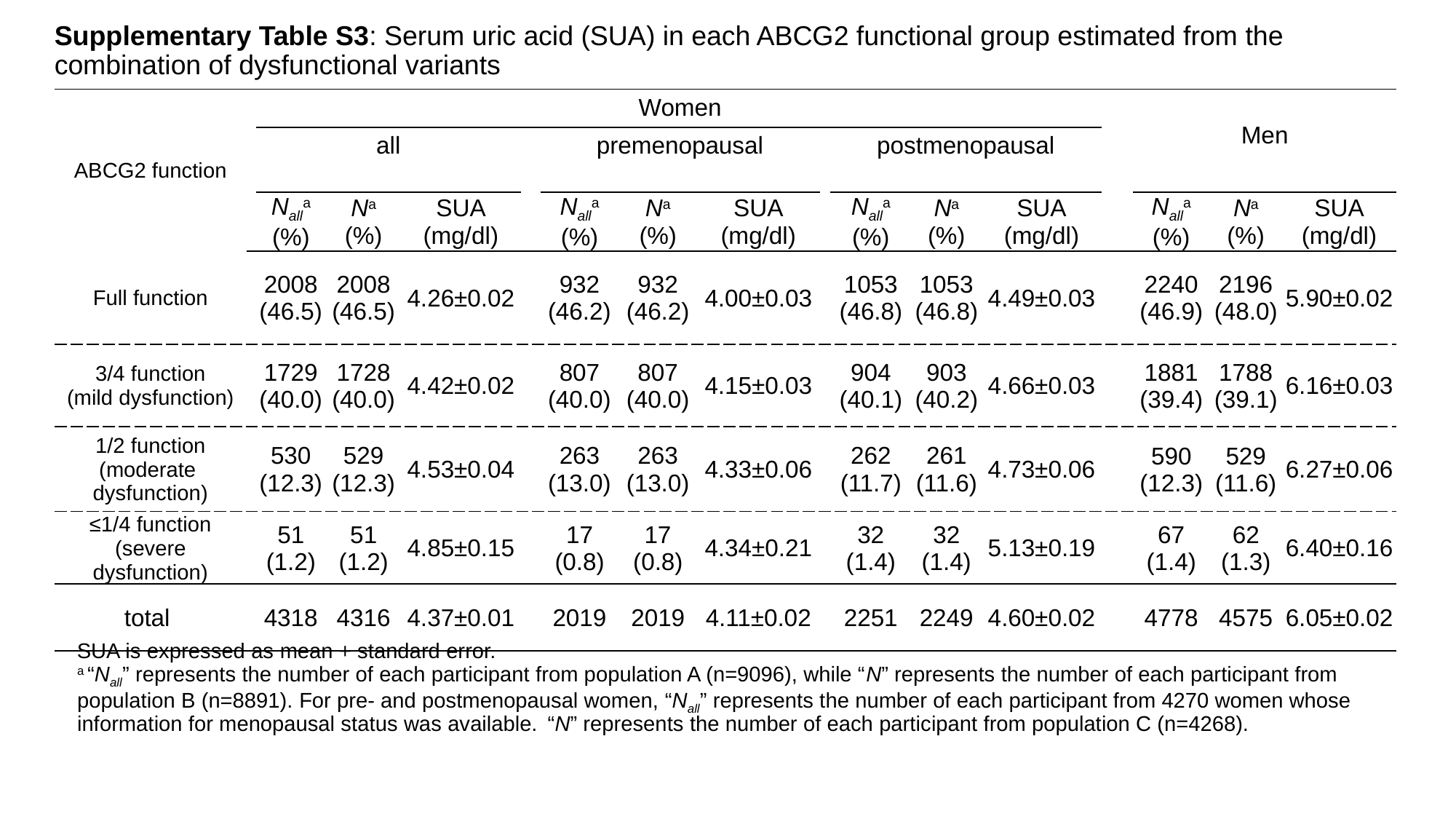

# Supplementary Table S3: Serum uric acid (SUA) in each ABCG2 functional group estimated from the combination of dysfunctional variants
| ABCG2 function | | | | | | Women | | | | | | | | Men | | |
| --- | --- | --- | --- | --- | --- | --- | --- | --- | --- | --- | --- | --- | --- | --- | --- | --- |
| | | all | | | | premenopausal | | | | postmenopausal | | | | | | |
| | | Nalla (%) | Na (%) | SUA (mg/dl) | | Nalla (%) | Na (%) | SUA (mg/dl) | | Nalla (%) | Na (%) | SUA (mg/dl) | | Nalla (%) | Na (%) | SUA (mg/dl) |
| Full function | | 2008 (46.5) | 2008 (46.5) | 4.26±0.02 | | 932 (46.2) | 932 (46.2) | 4.00±0.03 | | 1053 (46.8) | 1053 (46.8) | 4.49±0.03 | | 2240 (46.9) | 2196 (48.0) | 5.90±0.02 |
| 3/4 function (mild dysfunction) | | 1729 (40.0) | 1728 (40.0) | 4.42±0.02 | | 807 (40.0) | 807 (40.0) | 4.15±0.03 | | 904 (40.1) | 903 (40.2) | 4.66±0.03 | | 1881 (39.4) | 1788 (39.1) | 6.16±0.03 |
| 1/2 function (moderate dysfunction) | | 530 (12.3) | 529 (12.3) | 4.53±0.04 | | 263 (13.0) | 263 (13.0) | 4.33±0.06 | | 262 (11.7) | 261 (11.6) | 4.73±0.06 | | 590 (12.3) | 529 (11.6) | 6.27±0.06 |
| ≤1/4 function (severe dysfunction) | | 51 (1.2) | 51 (1.2) | 4.85±0.15 | | 17 (0.8) | 17 (0.8) | 4.34±0.21 | | 32 (1.4) | 32 (1.4) | 5.13±0.19 | | 67 (1.4) | 62 (1.3) | 6.40±0.16 |
| total | | 4318 | 4316 | 4.37±0.01 | | 2019 | 2019 | 4.11±0.02 | | 2251 | 2249 | 4.60±0.02 | | 4778 | 4575 | 6.05±0.02 |
SUA is expressed as mean + standard error.
a “Nall” represents the number of each participant from population A (n=9096), while “N” represents the number of each participant from population B (n=8891). For pre- and postmenopausal women, “Nall” represents the number of each participant from 4270 women whose information for menopausal status was available. “N” represents the number of each participant from population C (n=4268).

## Slide 5
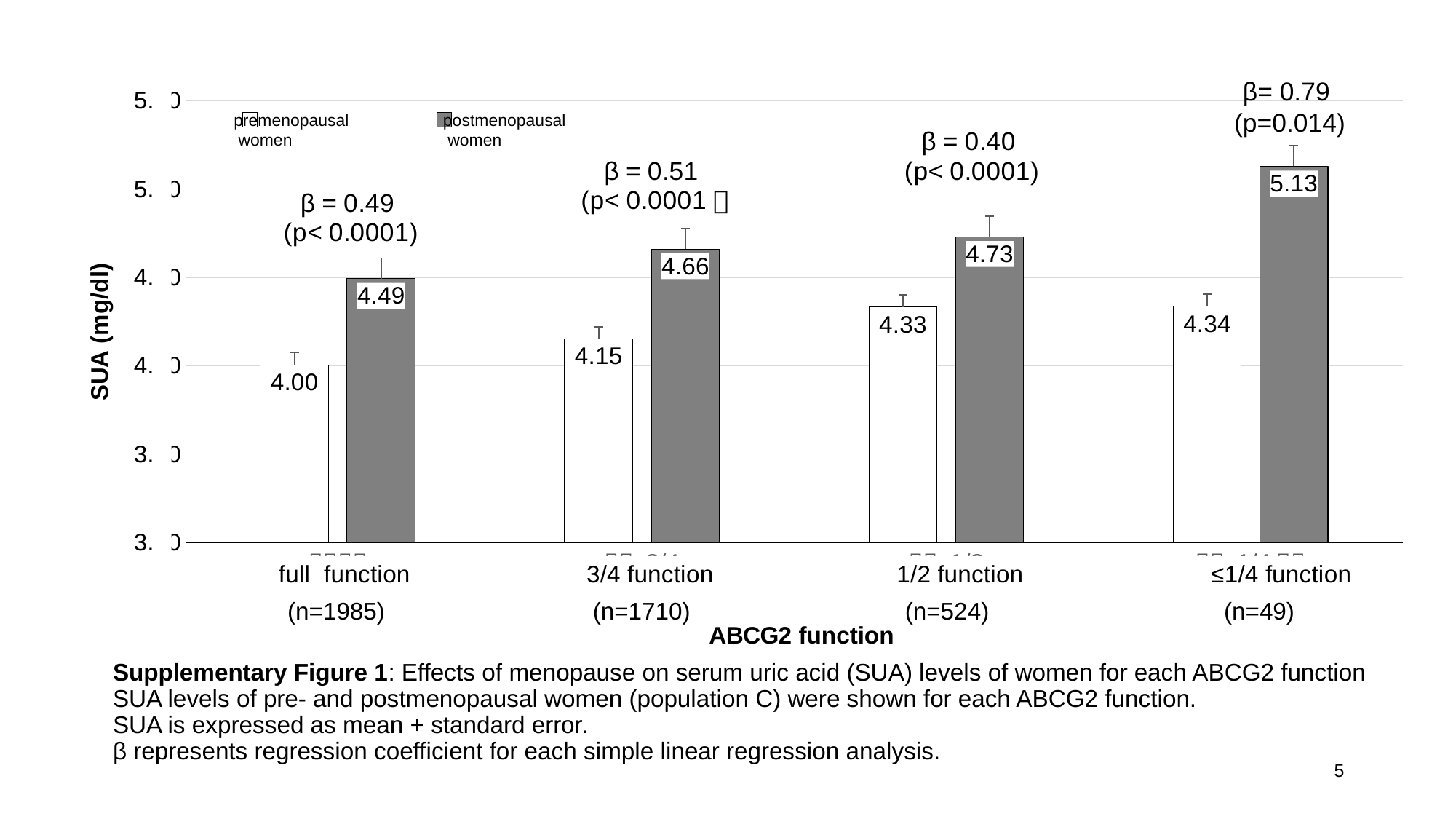

### Chart
| Category | 閉経前女性 | 閉経後女性 |
|---|---|---|
| 機能正常 | 4.0032189 | 4.4924976 |
| 機能 3/4 | 4.1510273 | 4.6601329 |
| 機能 1/2 | 4.3311787 | 4.7295019 |
| 機能 1/4以下 | 4.3352941 | 5.128125 |
β= 0.79
 (p=0.014)
premenopausal postmenopausal
 women women
 (n=1985) 　 (n=1710) (n=524) (n=49)
Supplementary Figure 1: Effects of menopause on serum uric acid (SUA) levels of women for each ABCG2 function
SUA levels of pre- and postmenopausal women (population C) were shown for each ABCG2 function.
SUA is expressed as mean + standard error.
β represents regression coefficient for each simple linear regression analysis.
5

## Slide 6
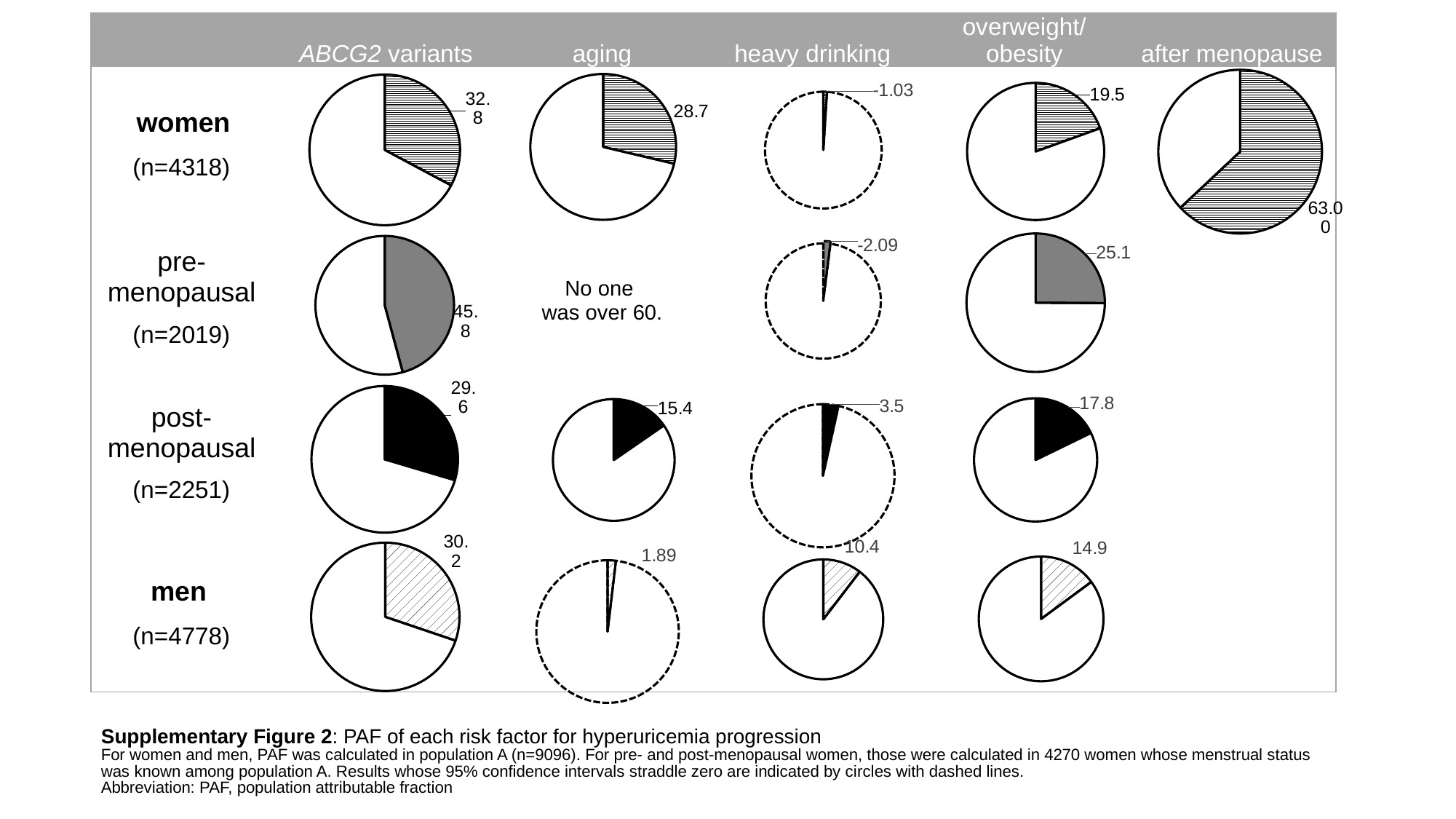

| | ABCG2 variants | aging | heavy drinking | overweight/ obesity | after menopause |
| --- | --- | --- | --- | --- | --- |
| women (n=4318) | | | | | |
| pre- menopausal (n=2019) | | No one was over 60. | | | |
| post- menopausal (n=2251) | | | | | |
| men (n=4778) | | | | | |
### Chart
| Category | mens |
|---|---|
### Chart
| Category | Overweight/obesity |
|---|---|
### Chart
| Category | Aging |
|---|---|
### Chart
| Category | ABCG2 variants |
|---|---|
### Chart
| Category | Heavy drinking |
|---|---|
### Chart
| Category | Heavy drinking |
|---|---|
### Chart
| Category | ABCG2 variants |
|---|---|
### Chart
| Category | Overweight/obesity |
|---|---|
### Chart
| Category | ABCG2 variants |
|---|---|
### Chart
| Category | Aging |
|---|---|
### Chart
| Category | Heavy drinking |
|---|---|
### Chart
| Category | Overweight/obesity |
|---|---|
### Chart
| Category | Aging |
|---|---|
### Chart
| Category | ABCG2 variants |
|---|---|
### Chart
| Category | Overweight/obesity |
|---|---|
### Chart
| Category | Heavy drinking |
|---|---|# Supplementary Figure 2: PAF of each risk factor for hyperuricemia progression
For women and men, PAF was calculated in population A (n=9096). For pre- and post-menopausal women, those were calculated in 4270 women whose menstrual status was known among population A. Results whose 95% confidence intervals straddle zero are indicated by circles with dashed lines.
Abbreviation: PAF, population attributable fraction
